# Tetra­kis{1-[4-(1*H*-imidazol-1-yl-κ*N* ^3^)phen­yl]ethanone}bis­(isothio­cyanato-κ*N*)nickel(II)

**DOI:** 10.1107/S1600536812027092

**Published:** 2012-06-23

**Authors:** Juan Zhao, Bao-Cheng Liu

**Affiliations:** a College of Mechanical Engineering, Qingdao Technological University, Qingdao 266033, People’s Republic of China; b Key Laboratory of Advanced Materials, Qingdao University of Science and Technology, Qingdao 266042, People’s Republic of China

## Abstract

The title complex mol­ecule, [Ni(NCS)_2_(C_11_H_10_N_2_O)_4_], has a crystallographically imposed centre of symmetry. The Ni^II^ atom is coordinated by the N atoms of two *trans*-arranged NCS^−^ anions and four 1-[4-(1*H*-imidazol-1-yl)phen­yl]ethan­one ligands in a distorted octa­hedral geometry. In the crystal, C—H⋯S hydrogen bonds link the complex mol­ecules into chains parallel to the *b* axis. The chains are further connected by C—H⋯O hydrogen bonds, forming layers parallel to the *bc* plane.

## Related literature

For the structures of related compounds, see: Liu *et al.* (2005 ▶, 2006 ▶); Pang *et al.* (2007 ▶); Zheng & Jin (2012 ▶).
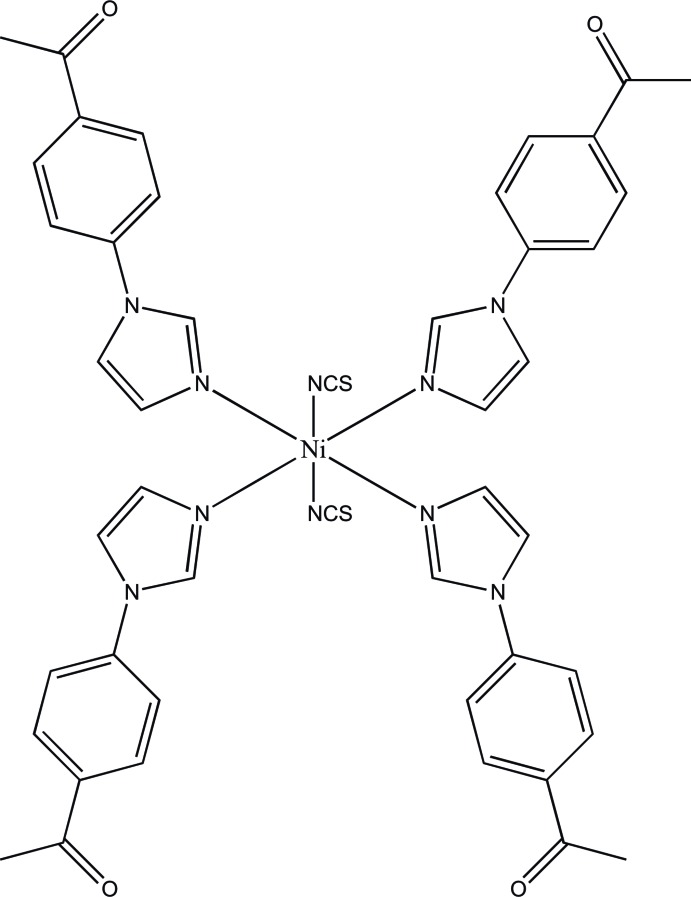


## Experimental

### 

#### Crystal data

[Ni(NCS)_2_(C_11_H_10_N_2_O)_4_]
*M* *_r_* = 919.71
Triclinic, 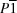

*a* = 8.4816 (4) Å
*b* = 8.8834 (4) Å
*c* = 15.0357 (8) Å
α = 85.701 (1)°
β = 88.161 (2)°
γ = 73.684 (1)°
*V* = 1084.12 (9) Å^3^
*Z* = 1
Mo *K*α radiation
μ = 0.60 mm^−1^
*T* = 293 K
0.18 × 0.12 × 0.08 mm

#### Data collection

Rigaku R-AXIS SPIDER diffractometer
Absorption correction: multi-scan (*ABSCOR*; Higashi 1995 ▶) *T*_min_ = 0.918, *T*_max_ = 0.951
8987 measured reflections
4005 independent reflections
2231 reflections with *I* > 2σ(*I*)
*R*_int_ = 0.057

#### Refinement

*R*[*F* ^2^ > 2σ(*F* ^2^)] = 0.065
*wR*(*F* ^2^) = 0.259
*S* = 1.09
4005 reflections
287 parameters
H-atom parameters constrained
Δρ_max_ = 0.65 e Å^−3^
Δρ_min_ = −1.22 e Å^−3^

### 

Data collection: *RAPID-AUTO* (Rigaku 2004 ▶); cell refinement: *RAPID-AUTO*; data reduction: *RAPID-AUTO*; program(s) used to solve structure: *SHELXS97* (Sheldrick, 2008 ▶); program(s) used to refine structure: *SHELXL97* (Sheldrick, 2008 ▶); molecular graphics: *SHELXTL* (Sheldrick, 2008 ▶); software used to prepare material for publication: *SHELXTL*.

## Supplementary Material

Crystal structure: contains datablock(s) global, I. DOI: 10.1107/S1600536812027092/rz2769sup1.cif

Structure factors: contains datablock(s) I. DOI: 10.1107/S1600536812027092/rz2769Isup2.hkl

Supplementary material file. DOI: 10.1107/S1600536812027092/rz2769Isup3.cdx

Additional supplementary materials:  crystallographic information; 3D view; checkCIF report

## Figures and Tables

**Table 1:** Hydrogen-bond geometry (Å, °)

*D*—H⋯*A*	*D*—H	H⋯*A*	*D*⋯*A*	*D*—H⋯*A*
C3—H3*A*⋯O1^i^	0.93	2.44	3.355 (8)	168
C9—H9*A*⋯S^ii^	0.93	2.87	3.759 (7)	160
C16—H16*A*⋯S^ii^	0.93	2.88	3.803 (8)	173

Symmetry codes: (i) 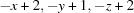; (ii) 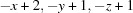.

**Fig. 1.**
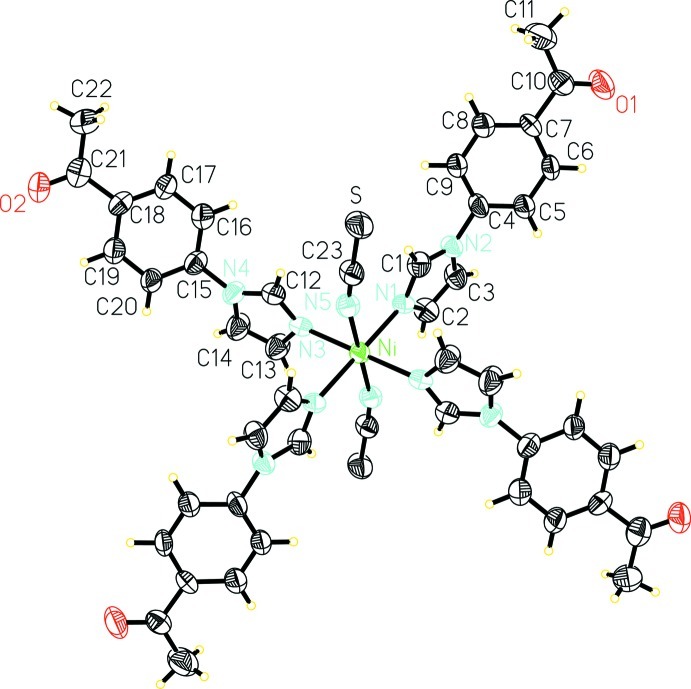
The structure of the title compound, showing 50% probability displacement ellipsoids. Unlabelled atoms are generated by the symmetry operation 2-x, 2-y, 1-z.

## supplementary crystallographic information

### Comment

Imidazole is of considerable interest as a ligand in many biological systems in which it provides a potential binding site for metal ions. Furthermore, the isothiocyanato anion is a versatile inorganic ligand in the synthesis of coordination compounds. As a continuation of our project devoted to study the conditions of the formation of thiocyanate-containing complexes with imidazole derivatives and to investigate the influence of steric properties on the stoichiometry of the resulting species (Liu *et al.*, 2005; Liu *et al.*, 2006; Pang, *et al.*, 2007; Zheng *et al.*, 2012), we report in the paper the crystal structure of the title compound.

The molecular structure of the title compound is shown in Fig. 1. The Ni atom lies on a centre of symmetry and displays a distorted octahedral coordination geometry, with the N atoms from two *trans*-arranged thiocyanate anions in the axial positions [Ni—N = 2.087 (5) Å] and the N atom of four 1-[4-(1*H*-imidazol-1-yl)phenyl]ethanone ligands at the equatorial plane [Ni—N = 2.097 (5)-2.125 (5) Å]. These values are in agreement with those ob served for the related compounds [Ni(NCS)_2_(1-methyl-1*H*-imidazole)_4_] (Liu, *et al.*, 2005), [Ni(NCS)_2_(1-ethyl-1*H*-imidazole)_4_] (Liu, *et al.*, 2006), [Ni(NCS)_2_(1-vinyl-1*H*-imidazole)_4_] (Pang, *et al.*, 2007), and [Ni(NCS)_2_(1-allyl-1*H*-imidazole)_4_] (Zheng, *et al.*, 2012). The equatorial N—Ni—N bond angles are close to those expected for a regular octahedral geometry [86.53 (18)–93.47 (18) °]. In the crystal structure, weak intermolecular C—H···S hydrogen interactions link the molecules into chains parallel to the *b* axis, which are further connected by C—H···O hydrogen bonds to form two-dimensional layers parallel to the *bc* plane.

### Experimental

The title compound was prepared by the reaction of 1-[4-(1*H*-imidazol-1-yl)phenyl]ethanone (3.72 g, 20 mmol) with NiSO_4_.6H_2_O (1.31 g, 5 mmol) and potassium thiocyanate (0.98 g, 10 mmol) by means of hydrothermal synthesis in stainless-steel reactor with Teflon liner at 393 K for 24 h. Single crystals suitable for X-ray measurements were obtained by slow evaporation of a methanol solution at room temperature. Analysis, calculated for C_46_H_40_NiN_10_O_4_S_2_: C 60.07, H 4.38, N 15.23%; found: C 60.21, H 4.34, N 15.36%.

### Refinement

H atoms were positioned geometrically (C—H = 0.93–0.96 Å) and allowed to ride on their parent atoms with *U*_iso_(H) = 1.2 *U*_eq_(C) or or 1.5 *U*_eq_(C) for methyl H atoms.

### Crystal data

**Table:**

[Ni(NCS)_2_(C_11_H_10_N_2_O)_4_]	*Z* = 1
*M**_r_* = 919.71	*F*(000) = 478
Triclinic, *P*1	*D*_x_ = 1.409 Mg m^−^^3^
Hall symbol: -P 1	Mo *K*α radiation, λ = 0.71073 Å
*a* = 8.4816 (4) Å	Cell parameters from 4970 reflections
*b* = 8.8834 (4) Å	θ = 6.6–54.9°
*c* = 15.0357 (8) Å	µ = 0.60 mm^−^^1^
α = 85.701 (1)°	*T* = 293 K
β = 88.161 (2)°	Block, blue
γ = 73.684 (1)°	0.18 × 0.12 × 0.08 mm
*V* = 1084.12 (9) Å^3^	

### Data collection

**Table:**

Rigaku R-AXIS SPIDER diffractometer	2231 reflections with *I* > 2σ(*I*)
Radiation source: fine-focus sealed tube	*R*_int_ = 0.057
Graphite monochromator	θ_max_ = 25.5°, θ_min_ = 3.3°
ω scans	*h* = −10→10
Absorption correction: multi-scan (*ABSCOR*; Higashi 1995)	*k* = −9→10
*T*_min_ = 0.918, *T*_max_ = 0.951	*l* = −18→18
8987 measured reflections	13 standard reflections every 0 reflections
4005 independent reflections	intensity decay: none

### Refinement

**Table:**

Refinement on *F*^2^	Secondary atom site location: difference Fourier map
Least-squares matrix: full	Hydrogen site location: inferred from neighbouring sites
*R*[*F*^2^ > 2σ(*F*^2^)] = 0.065	H-atom parameters constrained
*wR*(*F*^2^) = 0.259	*w* = 1/[σ^2^(*F*_o_^2^) + (0.1329*P*)^2^ + 1.2354*P*] where *P* = (*F*_o_^2^ + 2*F*_c_^2^)/3
*S* = 1.09	(Δ/σ)_max_ < 0.001
4005 reflections	Δρ_max_ = 0.65 e Å^−^^3^
287 parameters	Δρ_min_ = −1.22 e Å^−^^3^
0 restraints	Extinction correction: *SHELXL97* (Sheldrick, 2008), Fc^*^=kFc[1+0.001xFc^2^λ^3^/sin(2θ)]^-1/4^
Primary atom site location: structure-invariant direct methods	Extinction coefficient: 0.026 (6)

### Special details

**Table:**

Geometry. All e.s.d.'s (except the e.s.d. in the dihedral angle between two l.s. planes) are estimated using the full covariance matrix. The cell e.s.d.'s are taken into account individually in the estimation of e.s.d.'s in distances, angles and torsion angles; correlations between e.s.d.'s in cell parameters are only used when they are defined by crystal symmetry. An approximate (isotropic) treatment of cell e.s.d.'s is used for estimating e.s.d.'s involving l.s. planes.
Refinement. Refinement of *F*^2^ against ALL reflections. The weighted *R*-factor *wR* and goodness of fit *S* are based on *F*^2^, conventional *R*-factors *R* are based on *F*, with *F* set to zero for negative *F*^2^. The threshold expression of *F*^2^ > σ(*F*^2^) is used only for calculating *R*-factors(gt) *etc*. and is not relevant to the choice of reflections for refinement. *R*-factors based on *F*^2^ are statistically about twice as large as those based on *F*, and *R*- factors based on ALL data will be even larger.

### Fractional atomic coordinates and isotropic or equivalent isotropic displacement parameters (Å^2^)

**Table:**

	*x*	*y*	*z*	*U*_iso_*/*U*_eq_	
Ni	1.0000	1.0000	0.5000	0.0402 (4)	
S	1.2589 (3)	0.45967 (19)	0.44721 (13)	0.0642 (6)	
O1	1.2327 (8)	0.1316 (6)	1.0204 (3)	0.0820 (17)	
O2	0.3162 (8)	0.6234 (6)	0.0530 (3)	0.0815 (17)	
N1	0.9580 (6)	0.8862 (5)	0.6220 (3)	0.0434 (12)	
N2	0.9810 (6)	0.7107 (5)	0.7363 (3)	0.0447 (12)	
N3	0.7715 (6)	0.9988 (5)	0.4465 (3)	0.0414 (12)	
N4	0.6043 (6)	0.9326 (6)	0.3569 (4)	0.0487 (13)	
N5	1.1113 (7)	0.7805 (6)	0.4516 (3)	0.0493 (13)	
C1	1.0105 (8)	0.7372 (8)	0.6480 (4)	0.0518 (16)	
H1A	1.0618	0.6587	0.6106	0.062*	
C2	0.8867 (8)	0.9585 (7)	0.6959 (4)	0.0507 (16)	
H2A	0.8357	1.0657	0.6972	0.061*	
C3	0.9002 (8)	0.8533 (8)	0.7670 (4)	0.0532 (17)	
H3A	0.8623	0.8737	0.8248	0.064*	
C4	1.0289 (8)	0.5639 (7)	0.7894 (4)	0.0462 (15)	
C5	1.0964 (8)	0.5577 (7)	0.8703 (4)	0.0496 (16)	
H5A	1.1101	0.6488	0.8917	0.060*	
C6	1.1448 (8)	0.4186 (7)	0.9209 (4)	0.0508 (16)	
H6A	1.1906	0.4158	0.9766	0.061*	
C7	1.1257 (8)	0.2803 (7)	0.8896 (4)	0.0443 (15)	
C8	1.0581 (9)	0.2894 (8)	0.8055 (4)	0.0572 (18)	
H8A	1.0457	0.1987	0.7830	0.069*	
C9	1.0093 (8)	0.4304 (7)	0.7551 (4)	0.0509 (16)	
H9A	0.9641	0.4354	0.6990	0.061*	
C10	1.1798 (8)	0.1322 (8)	0.9466 (5)	0.0546 (17)	
C11	1.1688 (13)	−0.0185 (9)	0.9111 (6)	0.088 (3)	
H11A	1.2083	−0.1040	0.9550	0.131*	
H11B	1.2345	−0.0379	0.8577	0.131*	
H11C	1.0565	−0.0097	0.8978	0.131*	
C12	0.7482 (8)	0.8852 (7)	0.4024 (4)	0.0495 (16)	
H12A	0.8208	0.7849	0.4022	0.059*	
C13	0.6349 (8)	1.1254 (8)	0.4293 (5)	0.0598 (18)	
H13A	0.6173	1.2236	0.4518	0.072*	
C14	0.5321 (9)	1.0864 (8)	0.3760 (5)	0.065 (2)	
H14A	0.4313	1.1504	0.3557	0.078*	
C15	0.5460 (8)	0.8397 (7)	0.2991 (4)	0.0495 (16)	
C16	0.5548 (9)	0.6859 (8)	0.3247 (5)	0.0614 (19)	
H16A	0.5944	0.6438	0.3808	0.074*	
C17	0.5056 (9)	0.5935 (8)	0.2679 (5)	0.0572 (18)	
H17A	0.5145	0.4888	0.2853	0.069*	
C18	0.4430 (8)	0.6558 (7)	0.1852 (4)	0.0509 (16)	
C19	0.4326 (8)	0.8124 (8)	0.1608 (4)	0.0552 (17)	
H19A	0.3894	0.8558	0.1055	0.066*	
C20	0.4846 (8)	0.9049 (7)	0.2163 (4)	0.0510 (16)	
H20A	0.4787	1.0089	0.1986	0.061*	
C21	0.3882 (10)	0.5613 (9)	0.1216 (5)	0.066 (2)	
C22	0.4202 (12)	0.3878 (8)	0.1422 (6)	0.086 (3)	
H22A	0.3776	0.3435	0.0953	0.129*	
H22B	0.3673	0.3689	0.1976	0.129*	
H22C	0.5364	0.3398	0.1469	0.129*	
C23	1.1725 (8)	0.6465 (7)	0.4481 (4)	0.0470 (15)	

### Atomic displacement parameters (Å^2^)

**Table:**

	*U*^11^	*U*^22^	*U*^33^	*U*^12^	*U*^13^	*U*^23^
Ni	0.0483 (7)	0.0358 (6)	0.0356 (7)	−0.0097 (5)	−0.0031 (5)	−0.0029 (4)
S	0.0822 (14)	0.0385 (9)	0.0632 (12)	−0.0011 (9)	−0.0132 (10)	−0.0039 (8)
O1	0.128 (5)	0.067 (3)	0.045 (3)	−0.019 (3)	−0.024 (3)	0.009 (2)
O2	0.112 (5)	0.077 (4)	0.060 (3)	−0.032 (3)	−0.038 (3)	0.001 (3)
N1	0.055 (3)	0.026 (2)	0.048 (3)	−0.009 (2)	−0.007 (2)	−0.001 (2)
N2	0.056 (3)	0.039 (3)	0.037 (3)	−0.009 (2)	0.000 (2)	−0.002 (2)
N3	0.045 (3)	0.032 (2)	0.046 (3)	−0.010 (2)	0.002 (2)	0.003 (2)
N4	0.049 (3)	0.041 (3)	0.056 (3)	−0.010 (2)	−0.010 (3)	−0.009 (2)
N5	0.061 (3)	0.050 (3)	0.040 (3)	−0.020 (3)	0.001 (2)	−0.003 (2)
C1	0.064 (4)	0.056 (4)	0.037 (3)	−0.019 (3)	0.005 (3)	−0.013 (3)
C2	0.054 (4)	0.044 (3)	0.048 (4)	−0.004 (3)	0.008 (3)	−0.007 (3)
C3	0.061 (4)	0.058 (4)	0.036 (3)	−0.010 (3)	0.002 (3)	−0.006 (3)
C4	0.058 (4)	0.049 (4)	0.028 (3)	−0.011 (3)	−0.004 (3)	0.004 (3)
C5	0.060 (4)	0.049 (4)	0.039 (4)	−0.014 (3)	−0.007 (3)	0.000 (3)
C6	0.064 (4)	0.052 (4)	0.035 (3)	−0.013 (3)	−0.019 (3)	0.002 (3)
C7	0.049 (4)	0.043 (3)	0.036 (3)	−0.008 (3)	−0.002 (3)	0.011 (3)
C8	0.078 (5)	0.052 (4)	0.043 (4)	−0.021 (4)	−0.010 (3)	0.000 (3)
C9	0.067 (4)	0.048 (4)	0.038 (3)	−0.016 (3)	−0.012 (3)	0.000 (3)
C10	0.058 (4)	0.059 (4)	0.046 (4)	−0.019 (3)	0.006 (3)	0.005 (3)
C11	0.129 (8)	0.058 (5)	0.074 (6)	−0.024 (5)	−0.008 (5)	0.008 (4)
C12	0.054 (4)	0.043 (3)	0.052 (4)	−0.015 (3)	−0.004 (3)	0.003 (3)
C13	0.051 (4)	0.053 (4)	0.073 (5)	−0.009 (3)	−0.013 (4)	−0.010 (3)
C14	0.059 (4)	0.054 (4)	0.079 (5)	−0.007 (3)	−0.026 (4)	−0.009 (4)
C15	0.043 (4)	0.051 (4)	0.057 (4)	−0.016 (3)	−0.007 (3)	−0.009 (3)
C16	0.070 (5)	0.057 (4)	0.058 (4)	−0.018 (4)	−0.020 (4)	0.001 (3)
C17	0.068 (5)	0.054 (4)	0.054 (4)	−0.021 (3)	−0.016 (3)	−0.009 (3)
C18	0.049 (4)	0.051 (4)	0.059 (4)	−0.021 (3)	−0.005 (3)	−0.013 (3)
C19	0.059 (4)	0.059 (4)	0.048 (4)	−0.016 (3)	−0.012 (3)	−0.004 (3)
C20	0.053 (4)	0.044 (3)	0.054 (4)	−0.009 (3)	−0.018 (3)	−0.002 (3)
C21	0.070 (5)	0.075 (5)	0.058 (5)	−0.023 (4)	−0.007 (4)	−0.011 (4)
C22	0.117 (7)	0.053 (4)	0.094 (6)	−0.030 (5)	−0.028 (6)	−0.001 (4)
C23	0.059 (4)	0.047 (4)	0.032 (3)	−0.010 (3)	0.008 (3)	−0.007 (3)

### Geometric parameters (Å, º)

**Table:**

Ni—N5^i^	2.087 (5)	C6—H6A	0.9300
Ni—N5	2.087 (5)	C7—C8	1.392 (8)
Ni—N1	2.097 (5)	C7—C10	1.482 (8)
Ni—N1^i^	2.097 (5)	C8—C9	1.379 (8)
Ni—N3	2.125 (5)	C8—H8A	0.9300
Ni—N3^i^	2.125 (5)	C9—H9A	0.9300
S—C23	1.616 (7)	C10—C11	1.506 (10)
O1—C10	1.209 (8)	C11—H11A	0.9600
O2—C21	1.228 (8)	C11—H11B	0.9600
N1—C1	1.306 (7)	C11—H11C	0.9600
N1—C2	1.363 (8)	C12—H12A	0.9300
N2—C1	1.359 (8)	C13—C14	1.334 (9)
N2—C3	1.366 (8)	C13—H13A	0.9300
N2—C4	1.439 (7)	C14—H14A	0.9300
N3—C12	1.310 (8)	C15—C16	1.373 (9)
N3—C13	1.385 (8)	C15—C20	1.386 (8)
N4—C12	1.363 (8)	C16—C17	1.378 (9)
N4—C14	1.378 (8)	C16—H16A	0.9300
N4—C15	1.431 (7)	C17—C18	1.381 (9)
N5—C23	1.162 (8)	C17—H17A	0.9300
C1—H1A	0.9300	C18—C19	1.391 (9)
C2—C3	1.353 (9)	C18—C21	1.483 (9)
C2—H2A	0.9300	C19—C20	1.377 (9)
C3—H3A	0.9300	C19—H19A	0.9300
C4—C5	1.352 (8)	C20—H20A	0.9300
C4—C9	1.381 (8)	C21—C22	1.498 (10)
C5—C6	1.368 (8)	C22—H22A	0.9600
C5—H5A	0.9300	C22—H22B	0.9600
C6—C7	1.400 (8)	C22—H22C	0.9600
			
N5^i^—Ni—N5	180.0 (3)	C7—C8—H8A	119.4
N5^i^—Ni—N1	91.00 (19)	C8—C9—C4	118.7 (6)
N5—Ni—N1	89.00 (19)	C8—C9—H9A	120.6
N5^i^—Ni—N1^i^	89.00 (19)	C4—C9—H9A	120.6
N5—Ni—N1^i^	91.00 (19)	O1—C10—C7	120.8 (6)
N1—Ni—N1^i^	180.000 (1)	O1—C10—C11	120.3 (6)
N5^i^—Ni—N3	89.69 (19)	C7—C10—C11	118.9 (6)
N5—Ni—N3	90.31 (19)	C10—C11—H11A	109.5
N1—Ni—N3	93.47 (18)	C10—C11—H11B	109.5
N1^i^—Ni—N3	86.53 (18)	H11A—C11—H11B	109.5
N5^i^—Ni—N3^i^	90.31 (19)	C10—C11—H11C	109.5
N5—Ni—N3^i^	89.69 (19)	H11A—C11—H11C	109.5
N1—Ni—N3^i^	86.53 (18)	H11B—C11—H11C	109.5
N1^i^—Ni—N3^i^	93.47 (18)	N3—C12—N4	111.5 (6)
N3—Ni—N3^i^	180.000 (1)	N3—C12—H12A	124.3
C1—N1—C2	105.1 (5)	N4—C12—H12A	124.3
C1—N1—Ni	128.7 (4)	C14—C13—N3	110.3 (6)
C2—N1—Ni	125.7 (4)	C14—C13—H13A	124.9
C1—N2—C3	106.5 (5)	N3—C13—H13A	124.9
C1—N2—C4	127.7 (5)	C13—C14—N4	106.7 (6)
C3—N2—C4	125.7 (5)	C13—C14—H14A	126.7
C12—N3—C13	105.3 (5)	N4—C14—H14A	126.7
C12—N3—Ni	124.7 (4)	C16—C15—C20	120.4 (6)
C13—N3—Ni	128.0 (4)	C16—C15—N4	120.1 (6)
C12—N4—C14	106.3 (5)	C20—C15—N4	119.4 (6)
C12—N4—C15	125.6 (5)	C15—C16—C17	120.5 (6)
C14—N4—C15	128.1 (5)	C15—C16—H16A	119.7
C23—N5—Ni	162.1 (5)	C17—C16—H16A	119.7
N1—C1—N2	111.8 (6)	C16—C17—C18	120.3 (6)
N1—C1—H1A	124.1	C16—C17—H17A	119.9
N2—C1—H1A	124.1	C18—C17—H17A	119.9
C3—C2—N1	110.8 (5)	C17—C18—C19	118.5 (6)
C3—C2—H2A	124.6	C17—C18—C21	122.4 (6)
N1—C2—H2A	124.6	C19—C18—C21	119.1 (6)
C2—C3—N2	105.7 (6)	C20—C19—C18	121.7 (6)
C2—C3—H3A	127.1	C20—C19—H19A	119.1
N2—C3—H3A	127.1	C18—C19—H19A	119.1
C5—C4—C9	121.2 (5)	C19—C20—C15	118.5 (6)
C5—C4—N2	119.8 (6)	C19—C20—H20A	120.7
C9—C4—N2	119.0 (5)	C15—C20—H20A	120.7
C4—C5—C6	120.5 (6)	O2—C21—C18	121.0 (7)
C4—C5—H5A	119.8	O2—C21—C22	119.7 (7)
C6—C5—H5A	119.8	C18—C21—C22	119.3 (6)
C5—C6—C7	120.5 (5)	C21—C22—H22A	109.5
C5—C6—H6A	119.8	C21—C22—H22B	109.5
C7—C6—H6A	119.8	H22A—C22—H22B	109.5
C8—C7—C6	117.9 (5)	C21—C22—H22C	109.5
C8—C7—C10	123.4 (6)	H22A—C22—H22C	109.5
C6—C7—C10	118.7 (6)	H22B—C22—H22C	109.5
C9—C8—C7	121.1 (6)	N5—C23—S	177.9 (6)
C9—C8—H8A	119.4		
			
N5^i^—Ni—N1—C1	−172.5 (5)	C5—C6—C7—C10	180.0 (6)
N5—Ni—N1—C1	7.5 (5)	C6—C7—C8—C9	−0.8 (10)
N3—Ni—N1—C1	97.8 (5)	C10—C7—C8—C9	179.9 (6)
N3^i^—Ni—N1—C1	−82.2 (5)	C7—C8—C9—C4	0.1 (11)
N5^i^—Ni—N1—C2	−1.9 (5)	C5—C4—C9—C8	0.8 (11)
N5—Ni—N1—C2	178.1 (5)	N2—C4—C9—C8	178.9 (6)
N3—Ni—N1—C2	−91.6 (5)	C8—C7—C10—O1	−177.5 (7)
N3^i^—Ni—N1—C2	88.4 (5)	C6—C7—C10—O1	3.2 (10)
N5^i^—Ni—N3—C12	179.8 (5)	C8—C7—C10—C11	2.8 (10)
N5—Ni—N3—C12	−0.2 (5)	C6—C7—C10—C11	−176.5 (7)
N1—Ni—N3—C12	−89.2 (5)	C13—N3—C12—N4	0.6 (7)
N1^i^—Ni—N3—C12	90.8 (5)	Ni—N3—C12—N4	−164.2 (4)
N5^i^—Ni—N3—C13	18.5 (6)	C14—N4—C12—N3	−1.3 (7)
N5—Ni—N3—C13	−161.5 (6)	C15—N4—C12—N3	177.0 (6)
N1—Ni—N3—C13	109.5 (6)	C12—N3—C13—C14	0.3 (8)
N1^i^—Ni—N3—C13	−70.5 (6)	Ni—N3—C13—C14	164.4 (5)
N1—Ni—N5—C23	−14.2 (17)	N3—C13—C14—N4	−1.0 (9)
N1^i^—Ni—N5—C23	165.8 (17)	C12—N4—C14—C13	1.4 (8)
N3—Ni—N5—C23	−107.7 (17)	C15—N4—C14—C13	−176.8 (6)
N3^i^—Ni—N5—C23	72.3 (17)	C12—N4—C15—C16	44.8 (10)
C2—N1—C1—N2	−2.1 (7)	C14—N4—C15—C16	−137.4 (8)
Ni—N1—C1—N2	170.0 (4)	C12—N4—C15—C20	−133.6 (7)
C3—N2—C1—N1	1.7 (7)	C14—N4—C15—C20	44.3 (10)
C4—N2—C1—N1	−176.3 (6)	C20—C15—C16—C17	1.1 (11)
C1—N1—C2—C3	1.6 (8)	N4—C15—C16—C17	−177.2 (6)
Ni—N1—C2—C3	−170.7 (4)	C15—C16—C17—C18	−1.5 (12)
N1—C2—C3—N2	−0.6 (8)	C16—C17—C18—C19	0.6 (11)
C1—N2—C3—C2	−0.6 (7)	C16—C17—C18—C21	180.0 (7)
C4—N2—C3—C2	177.4 (6)	C17—C18—C19—C20	0.8 (11)
C1—N2—C4—C5	134.3 (7)	C21—C18—C19—C20	−178.7 (7)
C3—N2—C4—C5	−43.4 (9)	C18—C19—C20—C15	−1.2 (11)
C1—N2—C4—C9	−43.8 (9)	C16—C15—C20—C19	0.2 (10)
C3—N2—C4—C9	138.6 (7)	N4—C15—C20—C19	178.5 (6)
C9—C4—C5—C6	−1.0 (11)	C17—C18—C21—O2	171.7 (8)
N2—C4—C5—C6	−179.1 (6)	C19—C18—C21—O2	−8.9 (11)
C4—C5—C6—C7	0.3 (10)	C17—C18—C21—C22	−7.7 (11)
C5—C6—C7—C8	0.6 (10)	C19—C18—C21—C22	171.7 (8)

Symmetry code: (i) −*x*+2, −*y*+2, −*z*+1.

### Hydrogen-bond geometry (Å, º)

**Table:**

*D*—H···*A*	*D*—H	H···*A*	*D*···*A*	*D*—H···*A*
C3—H3*A*···O1^ii^	0.93	2.44	3.355 (8)	168
C9—H9*A*···S^iii^	0.93	2.87	3.759 (7)	160
C16—H16*A*···S^iii^	0.93	2.88	3.803 (8)	173

Symmetry codes: (ii) −*x*+2, −*y*+1, −*z*+2; (iii) −*x*+2, −*y*+1, −*z*+1.
